# Long-term effects of preeclampsia on maternal cardiovascular health and postpartum utilization of primary care: an observational claims data study

**DOI:** 10.1007/s00404-022-06561-w

**Published:** 2022-04-28

**Authors:** Kathrin Haßdenteufel, Mitho Müller, Raphael Gutsfeld, Maren Goetz, Armin Bauer, Markus Wallwiener, Sara Y. Brucker, Stefanie Joos, Miriam Giovanna Colombo, Sabine Hawighorst-Knapstein, Ariane Chaudhuri, Gudula Kirtschig, Frauke Saalmann, Stephanie Wallwiener

**Affiliations:** 1 Department of Obstetrics and Gynecology, Heidelberg University, Im Neuenheimer Feld 440, 69120 Heidelberg, Germany; 2 Department of Psychology, Ludwig Maximilian University, Munich, Germany; 3 Department of General Pediatrics, University Children’s Hospital, Heidelberg, Germany; 4 Department of Women’s Health, University Hospital Tuebingen, Tuebingen, Germany; 5 Institute for General Practice and Interprofessional Healthcare, Eberhardt-Karls-University, Tuebingen, Germany; 6 Department of Health Promotion, AOK Baden-Wuerttemberg, Stuttgart, Germany

**Keywords:** Preeclampsia, Cardiovascular disease, Hypertension, Pregnancy, Primary care

## Abstract

**Purpose:**

Preeclampsia occurs in up to 15% of pregnancies and constitutes a major risk factor for cardiovascular disease. This observational cohort study aimed to examine the association between preeclamptic pregnancies and cardiovascular outcomes as well as primary and specialized care utilization after delivery.

**Methods:**

Using statutory claims data we identified women with singleton live births between 2010 and 2017. Main outcomes included the occurrence of either hypertension or cardiovascular disease after one or more preeclamptic pregnancies, number of contacts to a general practitioner or cardiologist after delivery and prescribed antihypertensive medication. Data were analyzed using Cox proportional hazard regression models adjusted for maternal age, diabetes, dyslipidemia, and obesity.

**Results:**

The study cohort consisted of 181,574 women with 240,698 births. Women who experienced preeclampsia once had an increased risk for cardiovascular (hazard ratio, HR = 1.29) or hypertensive (HR = 4.13) events. In women affected by recurrent preeclampsia, risks were even higher to develop cardiovascular disease (HR = 1.53) or hypertension (HR = 6.01). In the following years after delivery, general practitioners were seen frequently, whereas cardiologists were consulted rarely (0.3 and 2.4%).

**Conclusion:**

Women affected by preeclampsia experience an increased risk of developing chronic hypertension and cardiovascular disease, especially those with recurrent preeclampsia. Future medical guidelines should take this potential risk into account.

**Supplementary Information:**

The online version contains supplementary material available at 10.1007/s00404-022-06561-w.

## Background

Pregnancy-related hypertensive disorders, including preeclampsia (PE) and gestational hypertension (GH), rank among the most common causes of maternal death and develop in up to 10% of pregnancies [1], with even higher rates of recurrence in up to 15% [2]. PE is defined either by new-onset hypertension and proteinuria after the 20th week of gestation (GW) or, alternatively, by the onset of PE-associated signs encompassing maternal organ dysfunction, even organ failure, or placental dysfunction [3, 4].

PE is presumably caused by placental hypoperfusion and hypoxia owing to abnormal development of the uterine placental spiral arteries. Hence, the emerging inflammatory response can cause endothelial dysfunction and vasoconstriction [5–7].

The pathophysiology of PE in its entirety is not fully understood and findings in the current literature diverge regarding mechanisms underlying future cardiovascular disease (CVD) [8, 9]. Widely discussed is the influence of factors such as obesity, diabetes, dyslipidemia, hypertension, a positive family history, and the occurrence of a metabolic syndrome [10–12]. Better known, however, are the diverse complications associated with PE, which, in turn, are due to the underlying vascular dysfunction [13, 14]. These include in particular placental insufficiency or abruption, intrauterine growth restriction, or intrauterine fetal death [15].

Most of the underlying pathological conditions and symptoms of pregnancy-related hypertensive disorders seem to resolve on their own shortly after birth [8]. Usually, spontaneous resolution of maternal hypertension and proteinuria is observed within the first week postpartum [8, 16].

Nevertheless, previous long-term studies have shown that hypertensive disorders during pregnancy are among the most important risk factors for cardiovascular diseases (CVD) [13, 17, 18]. Long-term cardiovascular adverse outcomes identified in women with a history of hypertensive disorders include the manifestation of chronic hypertension, ischemic heart disease, and stroke [19–21]. Consequently, both the American Heart Association (AHA) and the European Society of Cardiology (ESC) included hypertensive disorders during pregnancy as an official risk factor for CVDs [22, 23]. CVDs, in turn, constitute the leading causes of mortality in women and are responsible for 32–34% of deaths among women [24]. Thus, precautions and preventive measures in terms of lifestyle changes and minimizing underlying risk factors are of major importance [25, 26].

Despite the well-established risk association between a history of PE or GH and long-term complications, which present a major global health burden, current recommendations and guidelines regarding cardiovascular risk assessment, screening for high-risk patients, integrated care concepts for long-term follow-up or preventative intervention programs during the postpartum period are scarce [27]. The German Association of Gynecology and Obstetrics (DGGG) recommends a medical check-up for blood pressure and renal function parameters at three months postpartum and, in particular, a comprehensive explanation of potential risks for women shortly after giving birth [28]. However, the clinical implementation of these guidelines is still insufficient [29]. According to a nationwide study, half of the participating physicians were not even aware of the existing guidelines and did not offer structured follow-up advice for women at risk [8, 30]. Nevertheless, a recently published study has proven evidence for quality improvement in terms of postpartum compliance regarding clinical follow-ups and blood pressure regulation due to a bundled care concept for women with a history of PE [31].

The purpose of this large-scale cohort study is to investigate the risk for long-term cardiovascular maternal outcomes after both, first-time and recurrent PE using claims data from German statutory health insurance. Secondary goal of this study is to analyze the current utilization of primary and specialized care following a PE pregnancy. We hypothesize that postpartum care settings in Germany are insufficient regarding the probable considering impact on cardiovascular lifetime risk caused by preeclampsia.

## Materials and methods

### Data and study design

Data sets were obtained by searching claims data from the AOK Baden-Wuerttemberg, a major regional German statutory health insurance company, presently covering around 4.5 million people. Regularly insured women who delivered a live infant between January 1, 2010, and December 31, 2017, were included. We analyzed a subsample of mothers whose data could be matched with the data of their child/children (222,779 women with 291,091 births). Pregnancies with a history of hypertensive disorder were identified using ICD-10 codes (International Classification of Diseases). Women who developed CVD (including the entities listed in Table 1 below) or chronic hypertension after delivery were identified. Follow-up data were collected until September 30, 2019.

**Table 1:** List of ICD, DRG and OPS codes for disease definitions

	ICD-10 and ATC-codes	DRG codes
Delivery outcome	O36.4, O60.1, O60.2, O60.3, O75.7, O80, O81, O82, Z37.-, Z38.-	O01, O02, O60
Preeclampsia (exposure)	O14.-	None
Hypertension	I10.-, I11.-, I12.-, I13.-, I15.-	None
CVD	I20.-;I121; I22.-; I23.-; I24.-, I25.-, I51.6, I51.8; I51.9	None
Adiposity, overnutrition	E66.-, E67.8, E68, E78.-	None
Dyslipidemia	E78.-	None
Diabetes	E10.-, E11.-, E12.-, E13.-, E14.-,	None
Gestational diabetes	O24	None
Medication Diuretics Beta-blockers Calcium-channel blocker ACE inhibitors AT-II-receptor antagonist	C.- C03.- C07.- C08.- C09A.-, C09B C09C.-, C09D	None

Additionally, (Official Classification for Operations and Procedures) and DRG (Diagnosis Related Groups) OPS codes were used. A full list of ICD, OPS, and DRG codes is summarized in Table S1. Implausible delivery data were excluded (*n* = 964) from all analyses due to the miscoding of the quarter of birth in the claims data. We also excluded births of multiples (*n* = 5872) since they are generally associated with higher peripartum risks regarding fetal and maternal morbidity [32]. Moreover, births with a maternal age < 15 years (*n* = 23) were excluded as were subjects who were insured with the AOK for less than 40% of the observation period (*n* = 26,365). Births with preexisting maternal diagnoses of CVD (*n* = 6611) or hypertension (*n* = 12,747) (between January 1, 2007 and September 30, 2019) were excluded at baseline. Overall, *n* = 50,393 births were excluded (in some births more than one exclusion criterion applied) and the final sample consisted of 181,574 women with 240,698 births.

All study data were anonymized and cannot be traced to the study team. Patient identification numbers that were originally used for linking files within the insurance databases were encrypted. The study fulfills the eligibility criteria according to the STROBE guidelines for observational cohort studies. Ethical approval was obtained from the Ethics Committee of Tuebingen University Hospital and the University Medical Faculty.

### Study variables

#### Exposure variables

The main exposure of interest was a history of PE, as defined by the International Classification of Diseases (ICD-10 German modification see Table S1). PE presents one entity of pregnancy-related hypertensive disorders and should be distinguished from chronic and gestational hypertension, defined as the sole presence of hypertension after the 20th GW, as well as from isolated gestational proteinuria, defined as a protein-creatinine ratio > 30 mg/mmol in the absence of hypertension.

For the main analysis, we considered PE as a time-dependent variable so that a woman could contribute pregnancies and person-time to both unexposed and exposed groups during follow-up. Women were considered “unexposed” (1) if they never had a PE pregnancy, or (2) from the date of their first delivery without prior PE until the date of their first PE pregnancy, irrespective of subsequent pregnancy outcomes.

Thus, we finally stratified the births into three strata according to the status of the PE prior risk exposure: (1) no exposure to PE, (2) exposure to one PE pregnancy, and (3) exposure to at least two PE pregnancies.

#### Outcome variables

The main outcome variables of interest encompass cardiovascular health outcomes following PE pregnancies that were identified using ICD-10 codes. The set of hospitalization endpoints regarding cardiovascular outcomes, including chronic hypertension as well as all clinically relevant diagnoses summarized under the term “cardiovascular disease”, can be found in Table 2. Chronic hypertension is defined as a persistent mean arterial blood pressure over 140/90 mmHg or higher.

**Table 2:** Outcome variables: ICD codes

Category	ICD codes
Hypertension	I10.-, I11.-, I12.-, I13.-, I15.-
CVD Angina pectoris Myocardial infarction Recurrent myocardial infarction Acute complications after myocardial infarction Acute and chronic ischemic heart disease Unspecified cardiovascular disease Other heart diseases	I20.-; I121; I22.-; I23.-; I24.-, I25.-, I51.6, I51.8; I51.9

#### Covariates

Potential confounders included: maternal age, diabetes (preexisting as well as gestational diabetes), dyslipidemia, and obesity. Maternal exposure to obesity encompassed adiposity, overnutrition, and dyslipidemia. Possible confounders were selected according to findings of previous research and expertise [33–35].

### Statistical analysis

The statistical analyses were performed using R version 4.0.2 and R-Studio v. 1.3.1056 for Windows (32/64 bit) [36]. The observation period started with the date of delivery and, hence, women were followed until they received one or more diagnoses of the respective ICD codes for CVD or hypertension. Other possible endpoints of the study period included death, migration, change to another insurance company, or end of the study period. Thus, in these survival data (i.e., data regarding the occurrence of time-dependent events and censored data), we aimed to compare risk groups (at least one risk exposure to PE) with a reference group (no risk exposure to PE), adjusted for defined binary and parametric covariates.

Cox proportional hazard regression was used to estimate hazard ratios (HRs) and 95% confidence intervals (CIs). In our proportional hazard model, the risk groups (ii & iii) were compared to the reference group of individuals who were not exposed to PE (i). The respective time scale was defined as a number of years postpartum.

The HRs are interpreted as relative risks: an HR = 1 meant that there was no difference in the event rate between a risk group and the reference. An HR > 1 or < 1 meant that the event-rate of a risk group was > 1 or < 1 times the event rate of the reference group, respectively. The model was adjusted for confounders, such as maternal age, diabetes or obesity, and dyslipidemia. We set the critical *α*-error to *α* ≤ 0.01. However, as the sample size was extensive, and thus the statistical power to detect even small effects (HRs ≈ 1) amounted near to 1 − β = 1.00, we cannot conclude that an effect had any substantial meaning only because of its statistical significance. Thus, in this study, we focused on the interpretation of the effect sizes, i.e., the HR’s.

Moreover, the distribution of visits to a general practitioner and/or a cardiologist in the outpatient setting, stratified by the occurrence of PE throughout the observation time after birth at the descriptive level.

## Results

### Sample characteristics

The study population consisted of 181,574 women with 240,698 singleton live births. Mean maternal age at delivery was 30.62 years (standard deviation, SD 5.26 years). The average inter-pregnancy-intervals amounted to 2.75 years (SD 1.24 years). Mean observation time was 4.74 years (SD = 2.27 years). Further characteristics of the study population are listed in Table 3.

**Table 3:** Sample characteristics

	*f*	%		*f*	%
*Number of births*			*Birth mode*		
1	125,930	52.32	C-Section	74,231	30.84
2	95,024	39.48	Vaginal	165,363	68.70
≥ 3	19,744	8.20	Undefined	78	0.03
Missing	0	0.00	Missing	1026	0.43
*Obesity/dyslipidemia*			*(Gestational) diabetes*		
False	196,170	81.50	False	199,119	82.73
True	44,528	18.50	True	41,579	17.27
Missing	0	0.00	Missing	0	0.00
*Preterms*			*Preeclampsia*		
False	225,035	93.49	False	217,954	90.55
True	15,663	6.51	True	22,744	9.45
Missing	0	0.00	Missing	0	0.00
*Cardiovascular disease*			*Hypertension*		
False	235,586	97.88	False	220,760	91.72
True	5112	2.12	True	19,938	8.28
Missing	0	0.00	Missing	0	0.00

We observed a proportion of 8.40% of women (*n* = 20,233) with a history of one PE pregnancy and 1.04% of women (n = 2511) with a recurrent diagnosis.

Regarding outcome variables in the timeframe between 2010 and 2019, at least one diagnosis (inpatient and outpatient setting) from the CVD group was detected in 5,112 (2.12%), and hypertension in 19,938 (8.28%) women (Table 4).

**Table 4:** Outcome case numbers stratified by occurrence of preeclampsia

	No PE	One PE pregnancy	Two or more PE pregnancies
Cardiovascular disease			
* f*	4418	597	97
%	2.03	2.95	3.86
Hypertension			
* f*	12,684	6009	1245
%	5.82	29.70	49.58

*f* = frequency; % = percentage of strata "No PE" *n* = 217,954; "One PE pregnancy" *n* = 20,233; "Two or more PE pregnancies" *n* = 2511

Mean age at initial diagnosis of a CVD was 30.47 years (SD = 5.59 years) and hypertension 30.45 years (SD = 5.59 years).

Regarding our three strata according to the status of the risk exposure, there were

1. 217,954 cases with no exposure to PE.
2. 20,233 cases with one PE pregnancy.
3. 2,511 cases with at least two PE pregnancies.

### Main analyses

**Fig. 1:**
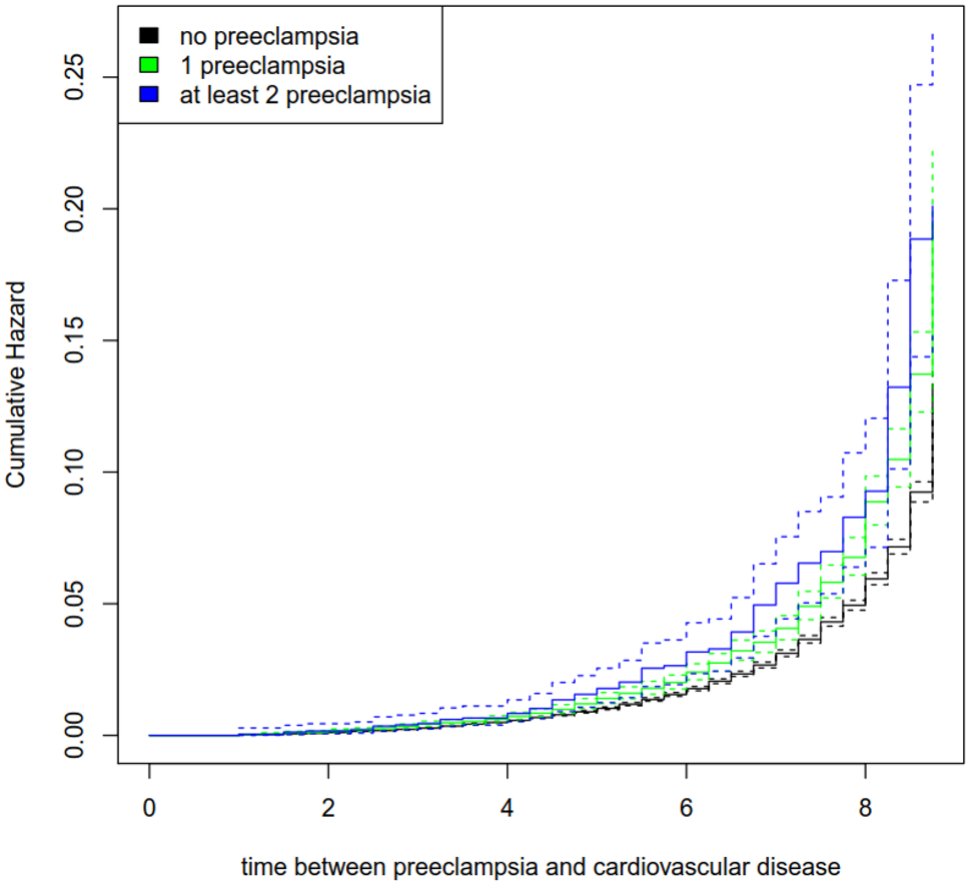
Cumulative hazard plot on the risk of any subsequent CVD

Compared to women with no risk exposure, women with a history of one PE pregnancy had a 28.8% increased risk of a subsequent CVD (see Table 5). For women, who had two or more PE pregnancies, the increased risk was 52.8%. The effect of obesity was comparable to the effects of the single PE exposures (HR = 1.384, see Table 3). The effects of maternal age and diabetes seemed low (maternal age: HR = 1.035; diabetes: HR = 1.151). The cumulative hazard plot regarding the risk of any subsequent CVD is depicted in Fig. 1.

**Table 5:** Cox regressions onto cardiovascular disease and chronic hypertension by risk exposures

Outcome	Exposure	HR	95% CI HR	*z*	*p*( >|*z*|)
Cardiovascular disease	One preeclampsia^d^	1.288	[1.181; 1.405]	5.727	< 0.001
	Two or more PE pregnancies^d^	1.528	[1.248; 1.873]	4.096	< 0.001
	Maternal age	1.035	[1.030; 1.040]	13.362	< 0.001
	Diabetes	1.151	[1.073; 1.236]	3.924	< 0.001
	Obesity	1.384	[1.298; 1.476]	9.866	< 0.001
Hypertension	One preeclampsia^d^	4.128	[4.000; 4.261]	88.080	< 0.001
	Two or more PE pregnancies^d^	6.007	[5.659; 6.376]	58.890	< 0.001
	Maternal age	1.060	[1.057; 1.063]	45.190	< 0.001
	Diabetes	1.353	[1.310; 1.397]	18.400	< 0.001
	Obesity	2.086	[2.025; 2.150]	48.110	< 0.001

*HR* hazard ratio, 95% *CI HR* 95% confidence interval of hazard ratio, *z z*-value, *p*( >|*z*|) empirical significance level

^a^*N* = 240,698

^b^Number of events = 5112

^c^Number of events = 19,938

^d^Reference group = no exposure to preeclampsia

**Fig. 2:**
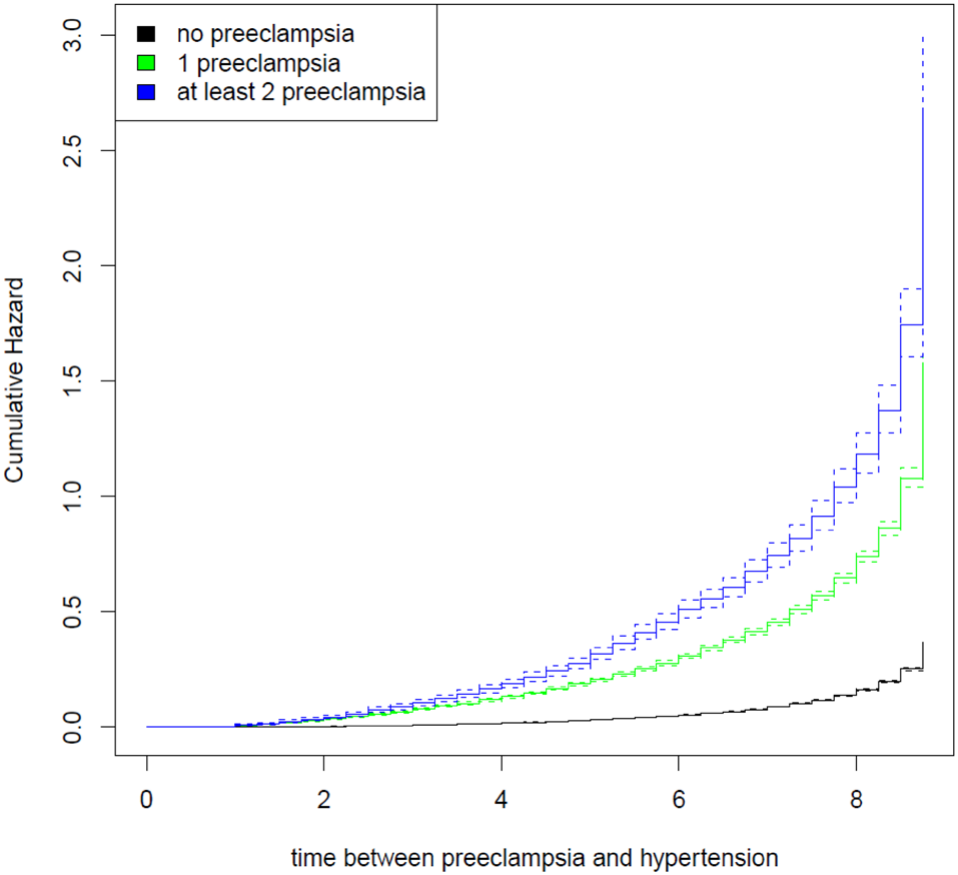
Cumulative hazard plot on the risk of subsequent hypertension

The effects of PE exceeded the respective effects from the prior model for the outcome of hypertension: compared to women with no risk exposure, women with a history of one PE pregnancy had a more than fourfold risk (i.e., an increase in risk by 312.8%) for subsequent, hypertension (see Table 3). For a history of at least two PE pregnancies, the risk was increased by 500.7%, which is more than a sixfold risk compared to women without any risk exposure. Maternal age, diabetes, and obesity had weaker effects, although the effects of diabetes and obesity seemed non-negligible (maternal age: HR = 1.060, diabetes: HR = 1.353, obesity: HR = 2.086) (Fig. 2).

### Additional analyses

#### Medical follow-up in women with a history of one or more PE pregnancies

**Fig. 3:**
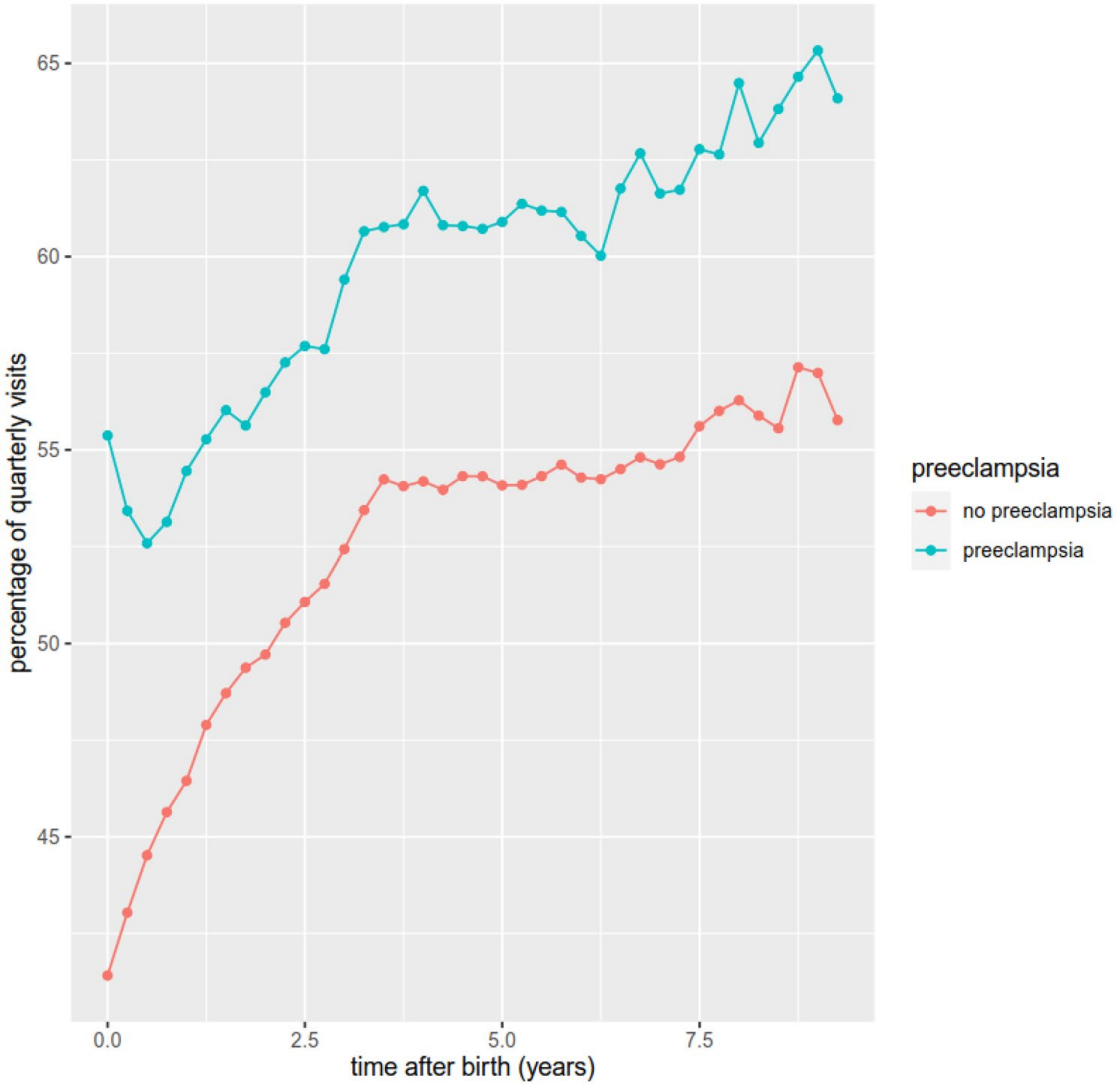
Visits to general practitioner stratified for the occurrence of PE

As shown in Fig. 3, the quarterly visits to a general practitioner mainly increase over time after birth. Between 41.4 and 65.3% of patients visit a general practitioner every quarter. The number of visits per quarter are increased in women who experienced a PE pregnancy by between 5.8 and 14.0%, with the highest gap immediately after birth.

**Fig. 4:**
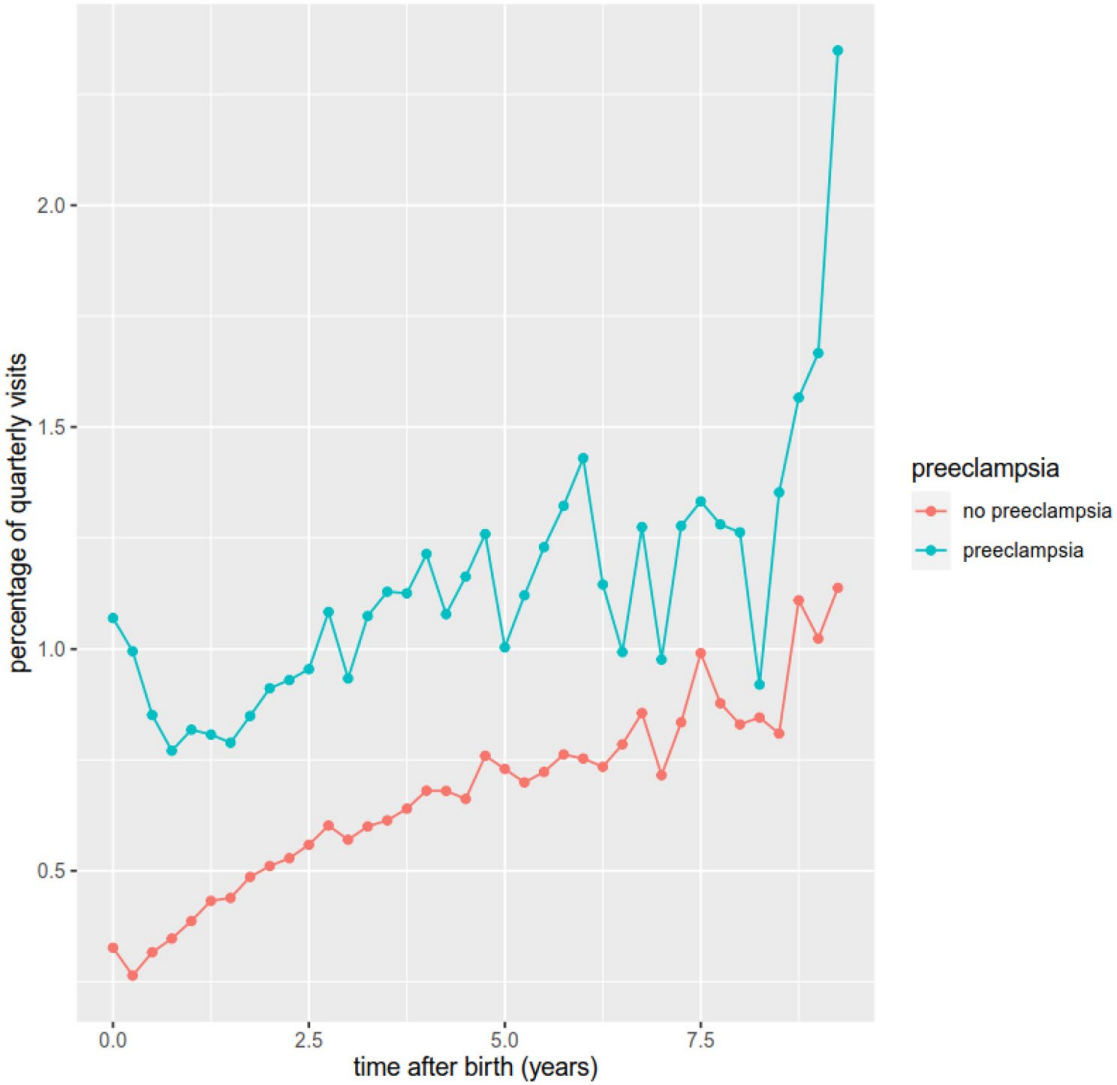
Visits to cardiologist stratified for the occurrence of PE

Accordingly, the number of quarterly visits to the cardiologist mainly increased by the time after the birth. However, the quarterly rates of visits to the specialist vary between 0.3 and 2.4% and are disproportionately lower than the rates of visits to the general practitioner. The rate of visits per quarter is generally increased in the group of women who experienced a PE pregnancy, varying between 0.1 and 1.2% with the highest gap at the end of the observation period at 9.25 years. Only 1% of patients who experienced a PE pregnancy consulted an internal specialist as recommended in the guidelines (Fig. 4) [28].

## Discussion

### Principal findings

To our knowledge, this observational, retrospective cohort study is the first to provide information on current postpartum disease incidence, care utilization, and medication intake in affected women. Our main analyses have shown that the occurrence of at least one PE pregnancy was significantly associated with a greater risk for cardiovascular adverse events and hypertension. The associated detrimental risk was even more pronounced when PE occurred in the second rather than in the first pregnancy. The adjusted risks ranged from 1.32 in one PE pregnancy to 2.03 in two or more for the outcome CVD. Regarding postpartum hypertension, we found HRs of 4.03 and up to 5.06 (two or more PE pregnancies). Thus, our findings are in accordance with previous research confirming PE to be a relevant risk factor for cardiovascular health in the long term and showing similar risk increases [25]. A large epidemiological review by Melchiorre et al. encompassing 87 publications emphasizes the elevated cardiovascular mortality as well and especially the urge for postpartum screening programs that should be initiated as early as possible [13]. In accordance with our findings, Magnussen et al. also observed a risk elevation according to the number of PE pregnancies [11]. Our study results also confirm those of Auger et al., who focused on recurrent PE and the risk of cardiovascular outcomes [37]. The authors also showed a decrease in the time period until the manifestation of the first cardiovascular event compared to women with no history of PE [38, 39].

Regarding postpartum follow-up, our data show that the relative number of medical check-ups decreases to a minimum shortly after giving birth for both general practitioner and cardiologist visits. The difference between affected and non-affected women remains negligible, especially in terms of specialized medical health care, which is rather unfavorable, as the postpartum period represents an important time frame in which an adequate risk management could significantly influence the risk for CVD in the long term [40]. In addition, previous research has shown a relatively low risk perception among women with a history of PE regarding cardiovascular outcomes in the long term [41]. Similar results have been published within the framework of the CVD prevention initiative in North America, showing that only 50% of referred women were seen by a cardiologist postpartum [42]. However, a scoping review published in 2019 reported that not only affected women but also health care providers lack in knowledge regarding long-term cardiovascular outcomes and the need for postpartum follow-up visits [30].

#### Perspectives

Our results suggest that women with a history of PE should be offered preventive counseling and lifestyle education in the postpartum period to improve access to early interventions and specialized care to optimize long-term outcomes.

### Strengths and limitations

As we used claims data in these analyses, the main limitation of our study is the missing adjustment regarding socioeconomic status and smoking. However, previous studies have shown that lifestyle factors do not constitute relevant risk factors for PE as compared to several genetic factors [43].

We could not assess the effect of timing regarding the onset of PE and the potentially different impacts of early- and late-onset PE. Moreover, further research could distinguish between different entities of pregnancy-related hypertensive disorders, including gestational hypertension, proteinuria, or preexisting hypertension, as they may lead to different long-term outcomes according to the current literature. Riise et al., however, found that long-term cardiovascular risks are similar for underlying PE and gestational hypertension, supporting the focus solely on PE in our work [44].

One of the strengths of our study, however, is that preexisting and known risk factors were excluded to examine PE as an independent risk factor insofar as possible. Furthermore, data were collected by insurance claims and not by self-report, which tends to over- or underrate results as diagnoses are stated in the context of billing purposes. The accuracy of diagnosis is guaranteed as far as possible by the use of medical records from a German insurance company. Hence, data could be collected in both the clinical and the outpatient setting, rendering our method of data collection far more reliable than that of other large studies based on self-report or merely on clinical data obtained from the hospital [45, 46]. The large sample size and the volume of observational claims data also render our findings highly precise and reduce potential sources of bias to a minimum.

Furthermore, we also focused on the current postpartum follow-up situation regarding the outpatient care setting in Germany to enhance the need for implementing feasible and cost-effective postpartum screening and developing prevention strategies for women at risk.

Our results are in line with the study of Heidrich et al., who showed that a large proportion of physicians are not aware of current recommendations regarding follow-up after giving birth. Less than 40% of the participating physicians even provided advice to reduce cardiovascular risk[47]. Thus, there is not only a need to establish an effective preventive program in high-risk populations but also to raise awareness among pregnant women and physicians alike regarding the burden of potential CVDs [48].

### Conclusion

Women with a history of PE pregnancies have a substantially higher risk of CVD and chronic hypertension in the long term. So far, there is still little knowledge and awareness among affected women and physicians regarding cardiovascular outcomes and existing recommendations for screening women at risk in Germany are scarce. Our findings emphasize the necessity for a structured postpartum disease and prevention management taking the potential adverse outcomes into account. Further research should focus on detecting women at high risk and evaluating the effectiveness of optimized primary care programs.

## Supplementary Information

Below is the link to the electronic supplementary material.

Supplementary file1 (PDF 84 kb)

## Data Availability

The data that support the findings of this study are available from AOK Baden-Wuerttemberg but restrictions apply to the availability of these data, which were used under license for the current study, and so are not publicly available. Data are however available from the authors upon reasonable request and with permission of AOK Baden-Wuerttemberg.

## Abbreviations

AHA: American heart association
ACE: Angiotensin-converting enzyme
AOK: Allgemeine Ortskrankenkasse (general local health insurance)
AT: Angiotensin
ATC: Anatomic therapeutic chemical classification system
CKD: Chronic kidney disease
CI: Confidence interval
CVC: Cardiovascular disease
DGGG: German association of gynecology and obstetrics
DRG: Diagnosis related groups
ESC: European society of cardiology
ESRD: End-stage renal disease
GH: Gestational hypertension
GW: Gestational week
HR: Hazard ratio
ICD: International classification of diseases
OPS: Operation and procedure classification system
PE: Preeclampsia
SD: Standard deviation
