# Supplementary material for: Long-term effects of preeclampsia on maternal cardiovascular health and postpartum utilization of primary care: an observational claims data study
Source: Arch Gynecol Obstet. 2022 Apr 28;307(1):275–84. doi: 10.1007/s00404-022-06561-w (PMC9836976; doi:10.1007/s00404-022-06561-w)
Supplement: Supplementary file 1 — Supplementary file1 (PDF 84 kb) [file 404_2022_6561_MOESM1_ESM.pdf]

Data Supplement

Table S1. *Visits to general practitioner stratified for the occurrence of PE.*

Time after birth (years)	No PE (%)	PE Pregnancies (%)	Difference (%)
0.00	41.41	55.37	13.96
0.25	43.04	53.42	10.39
0.50	44.52	52.58	8.06
0.75	45.64	53.13	7.50
1.00	46.45	54.45	8.01
1.25	47.89	55.27	7.38
1.50	48.71	56.03	7.31
1.75	49.37	55.63	6.26
2.00	49.71	56.49	6.78
2.25	50.54	57.26	6.72
2.50	51.07	57.69	6.61
2.75	51.54	57.61	6.06
3.00	52.43	59.41	6.97
3.25	53.44	60.66	7.22
3.50	54.24	60.77	6.53
3.75	54.06	60.84	6.78
4.00	54.19	61.70	7.52
4.25	53.97	60.81	6.84
4.50	54.32	60.79	6.47
4.75	54.31	60.72	6.41
5.00	54.08	60.90	6.82
5.25	54.09	61.37	7.27
5.50	54.32	61.19	6.87
5.75	54.62	61.16	6.54
6.00	54.28	60.54	6.26
6.25	54.24	60.02	5.78
6.50	54.50	61.76	7.26
6.75	54.81	62.67	7.86

7.00	54.63	61.63	7.01
7.25	54.82	61.73	6.91
7.50	55.61	62.78	7.16
7.75	56.01	62.64	6.63
8.00	56.28	64.49	8.21
8.25	55.88	62.94	7.06
8.50	55.56	63.82	8.26
8.75	57.14	64.65	7.52
9.00	56.99	65.33	8.34
9.25	55.77	64.09	8.33

Notes. PE = preeclampsia; % = percentage of quarterly visits in relation to observed patients

Table S2. *Visits to cardiologist stratified for the occurrence of PE.*

Time after birth (years)	No PE (%)	PE Pregnancies (%)	Difference (%)
0.00	0.33	1.07	0.74
0.25	0.26	1.00	0.73
0.50	0.32	0.85	0.54
0.75	0.35	0.77	0.42
1.00	0.39	0.82	0.43
1.25	0.43	0.81	0.37
1.50	0.44	0.79	0.35
1.75	0.49	0.85	0.36
2.00	0.51	0.91	0.40
2.25	0.53	0.93	0.40
2.50	0.56	0.95	0.40
2.75	0.60	1.08	0.48
3.00	0.57	0.93	0.36
3.25	0.60	1.07	0.47
3.50	0.61	1.13	0.52

3.75	0.64	1.13	0.49
4.00	0.68	1.21	0.53
4.25	0.68	1.08	0.40
4.50	0.66	1.16	0.50
4.75	0.76	1.26	0.50
5.00	0.73	1.00	0.27
5.25	0.70	1.12	0.42
5.50	0.72	1.23	0.51
5.75	0.76	1.32	0.56
6.00	0.75	1.43	0.68
6.25	0.73	1.15	0.41
6.50	0.79	0.99	0.21
6.75	0.86	1.27	0.42
7.00	0.72	0.98	0.26
7.25	0.83	1.28	0.44
7.50	0.99	1.33	0.34
7.75	0.88	1.28	0.40
8.00	0.83	1.26	0.43
8.25	0.85	0.92	0.07
8.50	0.81	1.35	0.54
8.75	1.11	1.57	0.46
9.00	1.02	1.67	0.64
9.25	1.14	2.35	1.21

Notes. PE = preeclampsia; % = percentage of quarterly visits in relation to observed patients

Table S3. *Prescription of hypertensive medication stratified for the occurrence of PE.*

Time after birth (years)	No PE (%)	PE pregnancies (%)	Difference (%)
0.00	0.58	10.62	10.04
0.25	0.40	7.41	7.01
0.50	0.45	7.28	6.83
0.75	0.48	7.14	6.67

1.00	0.50	7.41	6.91
1.25	0.55	7.66	7.10
1.50	0.60	7.81	7.20
1.75	0.66	8.12	7.46
2.00	0.72	8.56	7.84
2.25	0.83	9.23	8.39
2.50	0.98	9.76	8.79
2.75	1.08	10.45	9.37
3.00	1.13	11.45	10.33
3.25	1.25	12.32	11.07
3.50	1.36	13.07	11.72
3.75	1.54	13.83	12.30
4.00	1.62	13.82	12.20
4.25	1.79	15.15	13.36
4.50	1.86	14.33	12.47
4.75	2.01	14.67	12.66
5.00	2.25	16.49	14.24
5.25	2.41	17.48	15.08
5.50	2.48	16.63	14.15
5.75	2.78	18.81	16.02
6.00	2.85	19.67	16.82
6.25	2.89	19.03	16.14
6.50	3.00	19.67	16.67
6.75	3.30	18.43	15.12
7.00	3.64	20.28	16.64
7.25	3.82	22.79	18.97
7.50	3.92	24.13	20.21
7.75	4.05	21.26	17.21

Notes. PE = preeclampsia; % = percentage of quarterly visits in relation to observed patients
